# Stimulation of human damaged sperm motility with hydrogen molecule

**DOI:** 10.1186/s13618-014-0023-x

**Published:** 2015-01-10

**Authors:** Kumiko Nakata, Naoki Yamashita, Yoshihiro Noda, Ikuroh Ohsawa

**Affiliations:** Biological Process of Aging, Tokyo Metropolitan Institute of Gerontology, 35-2 Sakae-cho, Itabashi-ku, Tokyo, 173-0015 Japan; Reproductive Medicine Research Center, Yamashita Shonan Yume Clinic, 1-2-10 Kugenumaishigami, Fujisawa, 251-0025 Japan; Animal Facility, Tokyo Metropolitan Institute of Gerontology, 35-2 Sakae-cho, Itabashi-ku, Tokyo, 173-0015 Japan

**Keywords:** Frozen-thawed sperm, Hydrogen molecule, Male infertility, Mitochondria, Sperm motility

## Abstract

**Background:**

Sperm motility is a critical factor in male fertility. Low motility can be caused by a variety factors including abnormal spermatogenesis, oxidative damage, or depletion of intracellular ATP. Recent findings indicate that hydrogen molecule (H_2_) selectively reduces toxic reactive oxygen species. In this study, we investigated the effects of H_2_ on human sperm motility *in vitro*.

**Methods:**

Experimentally damaged sperm suspensions from patients left at room temperature for > 5 days or frozen immediately after ejaculation were used. After exposure with H_2_, their forward motility was measured with a counting chamber. A time-lapse movie was recorded to analyze sperm swimming speed. Mitochondria were stained with a membrane potential-sensitive dye.

**Results:**

H_2_ treatment significantly improved the rate of forward motility, whereas treatment with nitrogen gas did not. While treatment for 30 min was sufficient to improve motility, it did not affect sperm swimming speed. After 24 h, retreatment with H_2_ increased the motility again. H_2_ treatment also increased mitochondrial membrane potential. Forward motility of low motile frozen-thawed sperm from patients significantly improved with cleavage medium containing H_2_.

**Conclusions:**

Our results illustrated that H_2_ treatment stimulates low sperm motility. H_2_ is a new promising tool for male infertility treatments.

## Introduction

Several factors are present in infertile males with sperm function defects caused by asthenozoospermia [1]. Gene defects, including DNMT3B and MTHFR, have been well documented to correlate with this phenotype [2]. Mitochondria DNA haplogroups may affect sperm motility [3]. Systemic disorders such as polycystic kidney disease [4] also affect fertility and cause asthenozoospermia. Sperms are highly vulnerable to oxidative stress because they contain high concentrations of free unsaturated fatty acids, lack intracellular antioxidant enzymes, and have a limited capacity for DNA repair [5]. The precise mechanisms of motility loss in the sperm, the ability of this cell to fuse with the oocyte under oxidative stress, and the subsequent initiation of lipid peroxidation are not known [6]; however, both oxidative damage to the axoneme and depletion of intracellular ATP appear to be involved [7]. While mitochondria are crucial for ATP production, they are also the main source of reactive oxygen species (ROS), notably via the formation of superoxide in the electron transport chain. Nevertheless, low levels of ROS are essential and act as second messengers for the regulation of sperm functions [8].

Previous studies have examined the effects of seminal plasma levels or oral administration of zinc, aspartic acid or coenzyme Q_10_ on semen quality, and their effects *in vitro* [9-11]. Especially, myoinositol and xanthine derivates have turned out to be an effective tool in stimulation of sperm motility [12,13]. We reported previously that the hydrogen molecule (H_2_) dose-dependently reduces the hydroxyl radical (•OH) *in vitro*, whereas H_2_ is too weak to reduce physiologically important ROS such as NO• and superoxide [14]. H_2_, the smallest molecule in the universe, has the unique ability of rapidly diffusing across membranes; it can react with cytotoxic •OH in all organelles, including mitochondria and the nucleus, and thus effectively protect cells against oxidative damage. Indeed, H_2_ prevented a decrease in the cellular levels of ATP synthesized in mitochondria [14]. Many studies reported previously that H_2_ suppressed oxidative stress-induced injury in several organs, reduced ischemia-reperfusion injury in the brain, heart, liver, and retina [14-17], protected against nephrotoxicity [18], and suppressed radiation-induced acute injury in the lung [19]. In the present study, we used experimentally damaged sperm suspensions, and investigated whether H_2_ treatment exerts protective effects on human sperm. We further demonstrated the practical application of H_2_ treatment of frozen-thawed sperm from patients.

## Methods

### Preparation of sperm suspensions

Human sperm suspensions from donors were used in this study. This study was approved by the Institutional Review Board of Yamashita Shonan Yume Clinic with consent from patients receiving *in vitro* fertilization (IVF) treatment at the Yamashita Shonan Yume Clinic. All patients needed IVF and/or intracytoplasmic sperm injection (ICSI) because they showed seminal defects such as hypospermia, oligozoospermia and asthenozoospermia. All procedures followed were in accordance with the ethical standards of the responsible committee on human experimentation (institutional and national) and with the Helsinki Declaration of 1964 and its later amendments. Informed consent was obtained from all patients for being included in the study.

Semen suspensions from patients, who were asked to respect an ejaculatory abstinence period of 3–5 days, were collected by masturbation. A part of them were immediately frozen by the method described below. Sperm parameters were assessed according to World Health Organization criteria (2010) [20]. Collected samples were prepared by washing semen in cleavage medium (SAGE cleavage medium; CooperSurgical, CT, USA) supplemented with 10% plasma protein fraction (PPF; Baxter Healthcare, IL, USA) to remove seminal plasma, centrifuging through a two-layer Percoll® density gradient at 600 × *g* for 15 min, concentrating by centrifugation at 400 × *g* for 5 min, and resuspending sperm in cleavage medium with 10% PPF [21].

### Treatment of experimentally damaged sperm with hydrogen molecule

After IVF using sperm from patients, remaining sperms for discard were used. Each experimentally damaged sperm suspension left in room air at room temperature for > 5 days was divided into three groups as follows: untreated (i.e., control), H_2_-treated, and N_2_-treated. During experiments, sperm suspensions were kept in approximately atmospheric O_2_ concentration to enhance oxidative damage. A sperm suspension of 100 μL on a culture dish was placed into an exposure chamber (volume, approximately 5 L) with H_2_-mixed gases (5% CO_2_, 20% O_2_, 50% H_2_, and 25% N_2_) or N_2_-mixed gases (5% CO_2_, 20% O_2_, and 75% N_2_). After closing the exposure chamber tightly, these concentrations of mixed gases (1 L/min flow rate) were reached within approximately 5 min. To confirm saturation of the sperm suspensions with mixed gases, we monitored H_2_ and O_2_ concentrations with needle-type sensors (Unisense, Aarhus N, Denmark). After exposure to mixed gases, each sperm suspension was mixed well by pipetting and a 5–10 μL drop was placed in a counting chamber [22]. The sperm concentration, forward motility sperm rate, non-forward motility sperm rate, and immobility sperm rate were measured visually three times for each sperm suspension.

To evaluate the velocities of motile sperm, a drop of the suspension was placed in a counting chamber and time-lapse movies of sperm movement were recoded using a microscope (Olympus, Tokyo, Japan) for 10 s. Moving images were processed with ImageJ and the CASA (computer assisted sperm analysis) plugin [23]. Forward motility sperm were selected to calculate the velocity.

### Treatment of frozen-thawed sperm with hydrogen molecule

Equal amount of freshly prepared sperm suspension from 21 patients in cleavage medium and TEST-yolk buffer (Irvine Scientific, CA, USA) were mixed and dispended into cryotubes. After exposing to nitrogen steam for 5 minutes, cryotubes were stocked in liquid nitrogen [24]. To thaw frozen sperm solution, cryotubes were warmed at 37°C for 5 min, and then each frozen-thawed sperm suspension was dispensed into 4 vials. To prepare the sperm-wash medium containing H_2_, a 50, 75 or 100% of cleavage medium saturated with H_2_ was mixed with the medium equilibrated with 5% CO_2_. Sperm suspensions were washed for 5 minutes with them, and measured their motility.

### Fluorescent staining of sperm mitochondria

Mitochondria were co-stained with MitoTracker Green (MTG, 2 μM; Life Technologies, CA, USA) and tetramethylrhodamine methyl ester (TMRM, 2 μM; Life Technologies) for 30 min. MTG fluorescence was independent of the membrane potential; however, TMRM fluorescence was dependent on the membrane potential. MTG and TMRM were visualized with excitation at 488 and 543 nm, and emission at 510 and 565 nm with a laser-scanning confocal microscope (Leica, Wetzlar, Germany). Images were analyzed for the membrane potential of individual sperm using TMRM fluorescence intensity values [25]. Sperm viability was assessed by staining with propidium iodine (PI, 10 μM; Dojindo, Kumamoto, Japan) and Hoechst 33342 (10 μM; Dojindo). Stained sperm were visualized with excitation at 535 and 350 nm, and emission at 617 and 461 nm with a laser-scanning confocal microscope, respectively.

### Statistical analysis

All statistical analyses were conducted with JMP (SAS, NC, USA) and essentially performed using one-way ANOVA followed by a post hoc Dunnett's test and two-way ANOVA. Comparison of forward motility before and after treatment of each sperm solution was performed using the paired *t*-test. Differences between data were considered significant for *P*-values < 0.05.

## Results

### Improvement of experimentally damaged sperm motility by treatment with hydrogen molecule

**Figure 1:**
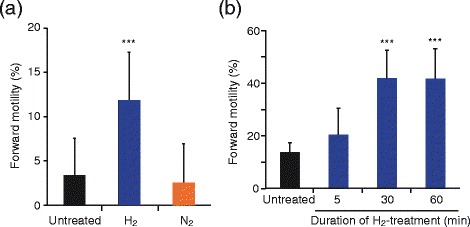
**Effect of H**_**2**_ **treatment on human sperm motility.** Damaged sperm suspensions (n = 35) were untreated or treated with H_2_- or N_2_-mixed gases for 40 min **(a)**. Another sperm suspensions (n = 12) were treated with H_2_-mixed gases for the indicated times **(b)**. Data are represented as means ± standard deviation (SD). ***P < 0.001, versus the untreated sperm suspension.

To assess the effect of H_2_ on sperm motility, we first prepared highly damaged sperms *in vitro*. Sperm suspensions from patients were left at room temperature for > 5 days, which enhanced oxidative stress and reduced sperm motility. The average of forward motility rate in 35 suspensions was 3.6% [90% confidence interval (CI): 0.6-6.7%]. We then treated the sperm suspensions with mixed gases containing 50% H_2_ gas (H_2_-mixed gases) for 40 min and found that the forward motility rate increased significantly (16.8%, 90% CI: 13.8-19.9%) (Figure 1a). On the other hand, the rate did not change (7.7%, 90% CI: 4.6-10.8%) after the sperm suspensions were treated with N_2_-mixed gases (without H_2_ gas). At that time, pH was almost equal between suspensions treated with N_2_- and H_2_-mixed gases (pH7.3 ± 0.1). Next, we examined the effect of treatment duration using other suspensions with relatively higher motility (n = 12). Both 30 and 60 min treatments with H_2_-mixed gas significantly improved sperm motility, whereas the 5 min treatment did not (Figure 1b), indicating that H_2_ treatment for 30 min is sufficient to improve motility.

### Effect of the sperm survival rate on H_2_ treatment

**Figure 2:**
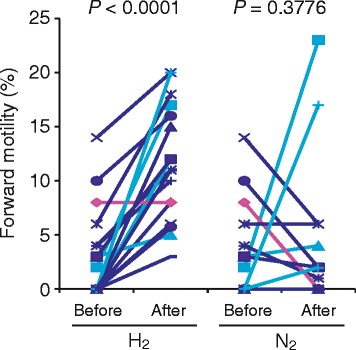
**Effect of H**_**2**_ **treatment on human sperm motility in each suspension.** Seventeen sperm suspensions with a sperm viability rate > 20% were used. Sperm motility was determined before and after treatment with H_2_- or N_2_-mixed gases for 30 min. Sperm motility in 12 suspensions (dark blue) improved only with H2-mixed gas, in 4 suspensions (light blue) improved with both H_2_- and N_2_-mixed gases and in one suspension (pink) was not activated by both mixed gases. *P*-values were determined using paired *t*-test.

The survival rate of sperm is different in each patient and affects the mobility [26]. We selected other sperm suspensions from 30 patients and examined survival by staining sperm with PI. The average percentage of PI-negative, viable sperm in all suspensions was ${18.6}_{-18.6}^{+35.0}\%$. We then divided them into two groups based on the percentage of viable sperm: high > 20% (n = 17, ${35.6}_{-12.0}^{+20.0}\%$) and low < 20% (n = 13, ${3.7}_{-3.7}^{+9.3}\%$). In low-viability group, sperm were immobile before and after treatment with mixed gases. However, we found that H_2_ treatment stimulated a very sluggish motility of several sperms with pendular movement of their heads around the axis of forward motility in 3 out of 13 suspensions, indicating that almost immobile sperm remaining a weak ability of movement may be activated by H_2_ treatment. We further investigated the effects of H_2_ treatment on sperm in high-viability group, in which the average forward motility was 3.0% (90.0% CI: 0.0-10.4%). After treatment with H_2_-mixed gas, we found that the forward motility in 16 suspensions significantly increased (Figure 2). Sperm motility in 4 suspensions improved with both H_2_- and N_2_-mixed gases, except for one suspension, which was not activated by both mixed gases. The average forward motilities in sperm suspensions after treatment with H_2_- and N_2_-mixed gases were 11.0% (90% CI: 2.7-20.0%) and 3.6% (90% CI: 0.0-17.6%), respectively. Thus, H_2_ treatment effectively and significantly improved sperm motility in suspensions with a higher survival rate.

### Effect of H_2_ treatment on sperm swimming speed

**Figure 3:**
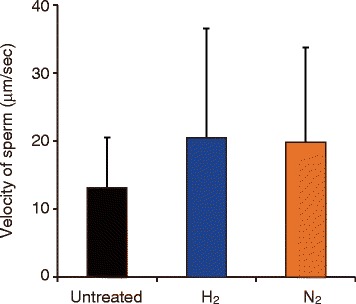
**Effect of H**_**2**_ **treatment on human sperm velocity.** Ten sperm suspensions were treated with H_2_- or N_2_-mixed gases for 30 min, and time-lapse movies of sperm motility were recorded with a microscope for 10 s. No significant difference was observed between control and treated groups. Data are represented as means ± standard deviation (SD).

To examine the effect of H_2_ treatment on sperm swimming speed, we used time-lapse microscopy and analyzed images with sperm analysis computer software, CASA. Ten sperm suspensions, which were randomly selected from 17 suspensions with a higher survival rate used in Figure 2, were treated with mixed gases for 30 min, and traveling distances of only moving sperm were measured for 10 s (Figure 3). No significant difference was observed between control and treated groups, indicating that H_2_ treatment does not affect sperm swimming speed.

### Retention time of H_2_ treatment on sperm motility

**Figure 4:**
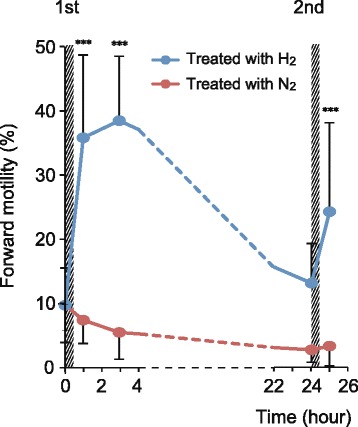
**Retention time of sperm suspensions with improved motility following H**_**2**_ **treatment.** Twelve sperm suspensions were treated with H_2_- or N_2_-mixed gases for 30 min (first treatment) and then retreated after 24 h, (second treatment), as indicated by hatching. Before and after the treatment, the suspension was kept at 25°C in room air. The average % of motile sperm per 1 mL of sperm suspension at the indicated times was calculated. Data represent means ± standard deviation (SD). ***P < 0.001; versus suspensions just before treatment (time = 0).

The retention time of motile sperm treated with H_2_ is an important indicator of IVF and ICSI success. After treatment with H_2_- or N_2_-mixed gases for 30 min, 10 sperm suspensions were kept at 25°C in room air. After 2.5 h, we found that forward motility in H_2_ treated suspension was still higher than that before treatment (Figure 4). It is noteworthy that H_2_ quickly diffuses. Approximately 10 min after placing an aliquot of the H_2_-treated sperm suspension in room air, the dissolved H_2_ concentration reached < 0.1% of its saturated concentration, indicating that the higher sperm motility enhanced by H_2_ treatment can be maintained without H_2_ for at least 2.5 h.

After the first H_2_ treatment, we further kept a sperm suspension at 25°C in room air for 24 h and found that its motility still remained a level approximately 36% of that of the first treatment (Figure 4). However, it was not significantly higher than that before treatment. We then treated this sperm suspension with H_2_ for 30 min (second treatment) and kept it at 25°C in room air for 30 min, and found that sperm motility increased again, reaching a level approximately 67% of that of the first treatment.

### Enhancement of mitochondrial membrane potential by H_2_ treatment

**Figure 5:**
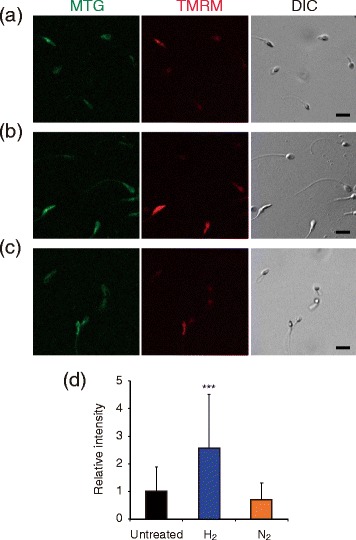
**Increase of mitochondrial membrane potential in sperm suspensions by H**_**2**_ **treatment.** Six sperm suspensions were either untreated **(a)**, or treated with H_2_- or N_2_-mixed gases (**b** and **c**, respectively), and stained with MitoTracker Green (MTG) and TMRM mitochondria- and mitochondrial membrane potential-sensitive fluorescent dyes. Differential interference contrast (DIC) images are also shown. Bars = 10 μm. The fluorescence intensity of each TMRM-stained sperm was semi-quantitatively analyzed by ImageJ **(d)**. Data represent means ± SD. ***P < 0.001, versus the untreated sperm suspension.

Since sperm motility is dependent on ATP content, we hypothesized that the improvement of sperm motility by H_2_ treatment might be due to changes in mitochondrial function. Therefore, after treatment with H_2_- or N_2_-mixed gases for 30 min by the same method described in Figures 1, 2 and 3, we stained sperm with TMRM, a mitochondrial membrane potential-dependent dye, and found that TMRM fluorescence increased (Figure 5), suggesting that H_2_ treatment may enhance mitochondrial function and sperm motility.

### Improvement in frozen-thawed sperm motility by treatment with hydrogen molecule

**Figure 6:**
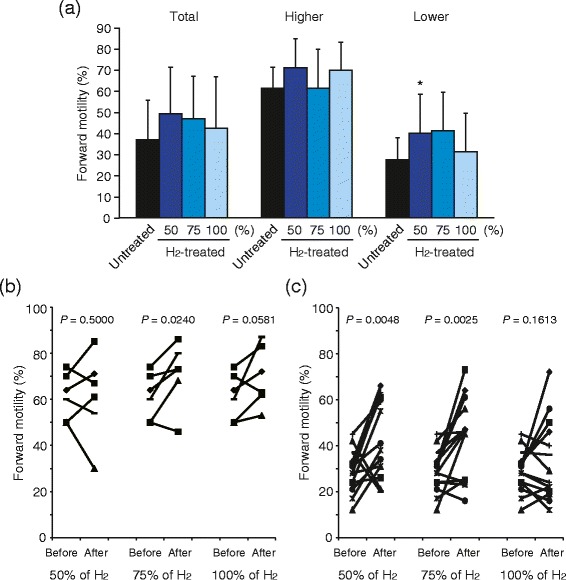
**Effect of H**_**2**_ **treatment on human frozen-thawed sperm motility in each suspension.** Total frozen-thawed sperm suspensions (n = 21) were divided into 2 groups, those with higher (forward motility ≥ 50%, normozoospermic, n = 6) or lower (<50%, asthenozoospermic, n = 15) motility. Sperm motility was determined before and after treatment with sperm-wash medium containing 50%, 75% and 100% of H_2_ for 30 min. Data represent means ± SD. *P < 0.05, versus the untreated sperm suspension **(a)**. Comparison of forward motility before and after treatment of each sperm suspension with higher **(b)** or lower **(c)** motility. *P*-values were determined using paired *t*-test.

To demonstrate the practical application of H_2_, we prepared frozen-thawed sperm suspension from each patient, which could be used for infertility treatment. The average forward motility of total 21 suspensions was 37.1% (95%CI: 28.7-45.6). Asthenozoospermia was defined according to the WHO guideline as samples with < 50% progressive sperm motility [20]. Therefore, we divided them into two groups, higher (forward motility ≥ 50%, n = 6) and lower (<50%, n = 15) suspensions, of which the average forward motilities before H_2_-treatment were 61.3% (95% CI: 50.8-71.8%) and 27.5% (95% CI: 21.7-33.2%), respectively. All suspensions were treated with sperm-wash media containing different concentrations (50, 75, and 100%) of H_2_. Dunnett's *t*-test for unpaired values indicated that the motility in suspensions of lower motile, asthenozoospermic sperms significantly increased after treatment with sperm-wash media containing 50% of H_2_, whereas treatment with 75% and 100% of H_2_ did not (Figure 6a). H_2_-treatment did not show any dose-dependency. The motility both in total and suspensions of higher motile, normozoospermic sperms did not significantly increase after H_2_-treatment. Next, we compared the forward motility before and after treatment of each sperm suspension by using the paired *t*-test. The forward motility in suspensions of higher motile sperms moderately increased after H_2_-treatment (Figure 6b). Treatments with 75% of H_2_ significantly improved the motility of suspensions, whereas treatments with 50% and 100% of H_2_ did not. On the other hand, we found that the forward motility in suspensions of lower motile sperms apparently increased after treatment with sperm-wash medium containing both 50% and 75% of H_2_ (Figure 6c), whereas treatment with 100% of H_2_ did not. These results indicated that H_2_-treatment effectively and significantly improved the motility of frozen-thawed sperms, especially low motile, asthenozoospermic ones.

## Discussion

Since previous studies have shown the balance between ROS and antioxidants to be unequivocally important for a variety of functions in the male reproductive system, we hypothesized that H_2_ treatment may be protective as a weak scavenger of ROS and may improve sperm motility *in vitro*. There are two reasons why sperm suspensions left at room temperature for > 5 days were used. Firstly, ejaculated sperm must be kept for 3 days at our clinic for infertility treatments, and remaining sperms for discard were used. Secondly, H_2_ is stable and must be coaxed with strong catalysts to enter into chemical reactions, and has been treated as an inactive gas in our body, indicating the possibility that the effects of H_2_ on sperm are not strong. Then, we expected that the using of highly damaged sperms was more sensitive to easily assess the effect of H_2_ on sperm motility.

The rate of forward motility increased from 3.6% to 16.8% (Figure 1a). The change in motility by H_2_ treatment was higher than or similar to results previously reported, e.g., 17.95% to 25.1% for treatment with platelet-activating factor [27] and a 21% increase for exogenous pyruvate treatment [28]. Because the increase of forward motility was observed within only 30 min, H_2_ treatment may stimulate cell signaling and activate mitochondria, but not transcription and translation in sperm. However, it remains to be elucidated whether the stimulatory effects are dependent on a property of H_2_ as a reductant. Furthermore, H_2_, but not N_2_, stimulated sperm motility, indicating that the effect of H_2_ did not rely on mechanical stimuli.

Effects were evident in almost all sperm suspensions (16 out of 17) with higher survival rates in which an increase of forward motility was observed with H_2_ treatment (Figure 2). Slight increase of the motility treated with N_2_ may due to the effect of mixing and shaking of sperm suspension during experimental manipulation [29]. The samples used in this study were collected from various patients with different physiological conditions and genetic backgrounds; therefore, it is possible that the effects of H_2_ rely on the character of each sperm suspension. The lack of response in forward motility in sperm suspensions with low survival rates indicated that H_2_ treatment was less effective on complete or nearly complete necrozoospermia. However, we found that H_2_ treatment stimulated a very sluggish motility of several sperms in 3 out of 13 suspensions with low survival rates, further indicating that H_2_ is beneficially effective on movement of sperms with both high and low survival rates. The differential effects of H_2_ treatment may be dependent on various conditions of sperm.

H_2_ did not enhance the sperm swimming speed (Figure 3), indicating that H_2_ did not hyperactivate sperm. Hyperactivation is characterized by a more energetic and less symmetric beat of sperm flagella and can be achieved *in vitro* by seminal plasma removal and incubation of sperm in capacitating medium. However, several reagents, including caffeine, which can stimulate sperm movement, have no effect on sperm velocity [30]. On the other hand, exogenous pyruvate accelerates glycolysis, and stimulates motility and hyperactivation with an increase in intracellular ATP levels [28]. It has been reported that glucose-derived ATP during capacitation involves hyperactivation [31], indicating that H_2_ treatment does not affect glycolysis.

We found that the higher sperm motility induced by H_2_ treatment was maintained for 2.5 h, even in the absence of H_2_ (Figure 4), which is clinically enough time for IVF and ICSI. The decrease in motility after H_2_ treatment is due to several reasons, including the reduction of intracellular ATP, and oxidative stress of oxygen in the room air [32]. Nevertheless, the motility increased the following day after a second H_2_ treatment. The increase of motility with second treatment of H_2_ was still higher than that of N_2_, indicating that the repeated increase was due to H_2_, but not mechanical stimuli. The repeated stimulation of sperm is likely to be useful in a clinical setting. Higher variation of the mobility in H_2_-treated sperm in Figure 4 may be dependent on its higher mobility, because the difference of coefficients of variation between the mobility of H_2_-treated sperm (0.26 to 0.59%) and that of N_2_ (0.48 to 0.98%) was very low.

A functional relationship among sperm mitochondrial membrane potential, sperm motility, and fertility potential has been proposed [33]. We found that H_2_ treatment enhanced mitochondrial membrane potential (Figure 5). Since sperm motility is dependent on ATP content, we speculate that H_2_ treatment enhances mitochondrial function, promotes ATP production, and then stimulates sperm motility. Indeed, we observed previously that H_2_ prevented a decrease in cellular levels of ATP synthesized in mitochondria [14]. Precise measurements of both ATP and calcium in sperm are needed before and after treatment with H_2_.

Finally, we used frozen-thawed sperm suspensions to validate the effects of H_2_ treatment on the motility of damaged sperm. Frozen sperm suspension is used routinely in assisted reproduction treatment. However, freezing has been reported to cause changes in sperm morphology, including damage to mitochondria. Sperm motility is particularly sensitive to freezing damage [34,35]. Effects were evident in 13 out of 15 suspensions of low motile frozen-thawed sperm (86.7%) in which an increase of forward motility was observed with sperm-wash medium containing 50% of H_2_ (Figure 6b), indicating that H_2_ treatment is clinically a potential approach to activate low motility sperm. However, treatment with medium containing higher concentration, 100%, of H_2_ was not effective, indicating the possibility that very low concentration of O_2_ in the medium might repress the sperm motility [36]. Further study is needed to elucidate a dose-dependency of H_2_-treatment.

The fertilization rates after IVF and ICSI using normal sperm are approximately 60% and 70%, respectively [37]. In general, the decision to perform either IVF or ICSI is dependent on sperm quality [38], which is determined by the total number of motile sperm [39]. While the concentration and morphology of ejaculated sperm do not affect ICSI results, the injection of a completely immotile spermatozoon is likely to have a negative effect on fertilization and the pregnancy rate [21,40]. Currently, there is no efficient therapy for asthenozoospermia.

## Conclusion

The findings of this study strongly indicate that H_2_ treatment activates low motility sperms. Importantly, recent studies demonstrated that H_2_ might have potential for wide use in medical applications as a novel, safe, effective antioxidant with minimal side effects [14-19]. We propose here that H_2_ is a new promising agent for male infertility treatment. However, to make practical use of H_2_ treatment, we further need to examine the effects of H_2_-treated sperm on fertilizing ability, embryonic development and safety for IVF and ICSI in future studies.

## Abbreviations

CI: Confidence interval
ICSI: Intracytoplasmic sperm injection
IVF: *In vitro* fertilization
MTG: MitoTracker Green
PPF: Plasma protein fraction
ROS: Reactive oxygen species
SD: Standard deviation
TMRM: Tetramethylrhodamine methyl ester
